# Comparative Analyses Reveal Mitogenome Characteristics of Halictidae and Novel Rearrangement (Hymenoptera: Apoidea: Anthophila)

**DOI:** 10.3390/ani15152234

**Published:** 2025-07-30

**Authors:** Dan Zhang, Zeqing Niu

**Affiliations:** 1 Characteristic Laboratory of Forensic Science in Universities of Shandong Province, Shandong University of Political Science and Law, Jinan 250014, China; zhangdan@sdupsl.edu.cn; 2 School of Criminal Justice, Shandong University of Political Science and Law, Jinan 250014, China; 3 State Key Laboratory of Animal Biodiversity Conservation and Integrated Pest Management, Institute of Zoology Chinese Academy of Sciences, Beijing 100101, China

**Keywords:** Nomiinae, mitochondrial genomes, gene rearrangement, phylogeny

## Abstract

**Simple Summary:**

Species of Halictidae are renowned for their outstanding pollination function and variable social behavior, while our understanding of the evolutionary history of this group has been hindered by the scarce molecular data. In this study, we newly obtained four mitogenomes of Halictidae. Using comprehensive comparative genomic methods, we analyzed the mitogenome base composition and codon usage of Halictidae which are similar to published bee species. We found five gene rearrangement patterns, including one novel pattern of bees. Furthermore, we combined published data to reconstruct phylogenetic relationships based on Bayesian Inference and Maximum Likelihood methods. Our study enriches molecular databases for research on bees and provides resources for further study on the evolutionary biology of Halictidae.

**Abstract:**

Halictidae, as a major pollinator family in bees, has significant ecological value. However, the insufficient molecular data for this group has limited our understanding of the evolutionary history of this group. Herein, we newly sequenced and assembled four mitogenomes of Halictidae, including three species of Nomiinae and one species of Rophitinae. We analyzed the characters of the newly obtained mitogenomes, including nucleotide composition, sequence length, and gene rearrangements. The length of the newly sequenced mitogenomes ranged from 16,492 to 21,192 bp, and all newly obtained mitogenomes contained 22 tRNAs, 13 protein-coding genes, two rRNAs, and one control region. Their AT content (%) ranged from 82.55 to 86.44. Relative synonymous codon usage analysis showed that UUU, UUA, and AUU were the preferred codons. The relative synonymous codon usage > 2 of mostly newly sequenced species was as follows: UUA > UCA > CGA. All newly obtained mitogenomes show gene rearrangement; we found five gene rearrangement patterns in total. Notably, *ND4-trnP-ND4L-trnT* was the first reported gene rearrangement pattern in bees. In addition, we reconstructed the phylogenetic relationships of Halictidae based on 10 species (eight ingroups and two outgroups), using Bayesian Inference and Maximum Likelihood approaches. Phylogenetic analysis showed that Rophitinae was the basal group within Halictidae.

## 1. Introduction

Among bees, the family Halictidae contains major pollinators, with more than 4400 described species [1,2,3]. Species of this group can be found in nearly all terrestrial environments; in many temperate areas of the world, halictids dominate other bees in number of individuals, except the species Apidae [3,4,5]. Halictidae contains four subfamilies, including Rophitinae, Nomiinae, Halictinae, and Nomioidinae [1,4].

Nomiinae, as one of the subfamilies within the family Halictidae, contains more than 620 described species; Asia and Africa are the hotspots of richness centers [2,3,4]. Nomiinae species hold significant value in agricultural economic development. Certain species within this group are actively managed for crop pollination [6]. For instance, *Nomia melanderi* serves as an effective pollinator of alfalfa and has been extensively utilized in the United States since the 1990s [6,7]. In addition, this species is the only solitary ground-nesting bee that is artificially managed [6,8,9]. Nevertheless, the studies of this group have predominantly concentrated on the description of new species or the review of a single genus or subgenus. Consequently, there is a significant deficiency in molecular data, and the phylogenetic relationships within Nomiinae remain inadequately understood. Michener, based on morphological characters, divided Nomiinae into 11 genera and 19 subgenera, which were supported by most bee researchers [10]. As research progressed, Baker, Pesenko, and Pauly suggested that the subgenera *Pseudapis*, *Nomiapis*, *Maynenoiay*, *Maculonomia*, *Gnathonomia*, and *Austronomia* need to be treated at the genus level [5,11,12]. However, Pauly mainly focused on the taxa of Africa and the Western Pacific, and both studies only used morphological characters without molecular data, but most bee researchers disagree with those points. Actually, there are significant differences among these subgenera in terms of morphological characteristics. For instance, certain species within *Gnathonomia* and *Maculonomia* lack colored bands on the apical margin of the metasomal terga [2,13], which does not conform to the diagnostic criteria of the genus *Nomia*. It is insufficient to rely on morphological characteristics; additional molecular data should be incorporated. However, only one published mitochondrial genome exists for the >600 species of Nomiinae (accessed on 20 May from NCBI database). Thus, the deficient molecular data of Nomiinae have seriously limited our understanding of the evolutionary history and phylogenetic relationships of this group, and also of Halictidae in general.

Rophitinae is a relatively small subfamily within Halictidae, with 261 described species [1]. Rophitinae is the only subfamily of Halictidae in which all members are solitary [4]. Species within Rophitinae exhibited restricted floral specialization, particularly the genus *Dufourea* [14,15]. *Dufourea* is a major genus of Rophitinae; species of this genus are nonmetallic black or dull greenish or bluish metallic, sometimes with the metasoma red [4]. Alves-dos-Santos (2003) observed that mouthparts of bees specializing in Pontederiaceae, such as *Dufourea novaeangliae*, have evolved hairs to remove pollen from the concealed anthers [16]. Despite the existence of some studies on this group, research on the mechanisms of molecular evolution remains limited. Notably, the available molecular data for this group are insufficient, constraining evolutionary research.

Mitochondrial genomes, as important molecular markers, have typically been employed in investigations pertaining to phylogeny, evolutionary history, speciation, and phylogeography within the taxonomic group of insects [17,18,19]. The maternal mode of inheritance, which is characterized by a high degree of substitution and the ease of obtaining these genomes, renders them particularly advantageous for the aforementioned research [20,21]. Insects' mitochondrial genomes typically span between 14,000 and 20,000 bp in length, encompassing two ribosomal RNAs (rRNAs), 22 transfer RNAs (tRNAs), 13 protein-coding genes (PCGs), and one non-coding control region (CR) [21]. Insects’ mitochondrial genomes have highly conserved composition and organization [21]. Furthermore, the structural characteristics of mitogenomes can offer additional insights and corroborating evidence for taxonomic classification [21,22]. In recent years, the number of complete mitochondrial genomes of insects that have been sequenced has increased, advancing the rapid development of next-generation sequencing technologies to resolve the structure comparison and evolutionary history for different groups [20,21,22,23,24].

Herein, we newly sequenced, assembled, and annotated four mitogenomes of the family Halictidae; all data have been submitted to the NCBI database. Combined with one published mitochondrial genome, we analyzed the main structure, evolutionary rate, and substitution of all newly obtained mitogenomes. In addition, we combined them with six published mitochondrial genomes of Halictidae to reconstruct the phylogenetic relationships of Halictidae based on Bayesian Inference (BI) and Maximum Likelihood (ML) approaches.

## 2. Materials and Methods

### 2.1. Taxon Sampling and Sequencing

Here, four bee species within Halictidae were newly sequenced and assembled (detailed information are shown in Table 1). All specimens were stored in 99% ethanol at −20 °C before species identification and DNA extraction. To compare the mitochondrial characters of the four newly sequenced species and also for the phylogenetic analyses, we downloaded one mitochondrial genome of *Nomia* (*Nomia chalybeata*) from GenBank. Two taxonomists (Dan Zhang and Zeqing Niu) identified all species based on morphological characteristics [2,3,4]. 

Legs of each sample were used to extract whole genome DNA with a Qiagen DNeasy Blood & Tissue Kit (Qiagen, Venlo, The Netherlands) and Qubit^®^ DNA Assay Kit in Qubit^®^ 2.0. A flurometer (ThermoFisher, Waltham, MA, USA) was used to measure the concentration of the whole genome DNA. The Illumina NovaSeq 6000 platform was used to generate sequencing libraries of 150 bp paired-end reads with an insert size of 350 bp. Short, adapter-contaminated, and low-quality reads of raw data were removed via Trimmomatic v0.32 [25].

### 2.2. Assembly, Annotation, and Composition Analyses

We used two different methods to assemble the mitogenome: (1) Illumina reads were assembled by NOVOPlasty v3.8.3 (Brussel, Belgium) [26] and the k-mer sizes of 23–39 bp; (2) High-quality reads were used for assembly via IDBA-UD v1.1.3 (Boston, MA, USA) [27], while the minimum and maximum k-values were 40 and 120 bp, respectively. Geneious 2020.2.1 [28] was performed to compare mitogenome sequences, which were obtained by the above methods and then merged into a single sequence. tRNAscan SE [29] was used for the secondary structure analysis of tRNAs. Clustal Omega in Geneious (2020.2.1) was applied to annotate the rRNAs and PCGs. MEGA X [30] was performed to check the boundaries of PCGs and rRNAs. SeqKit v0.16.0 (Chongqing, China) [31] was implemented to check the nucleotide composition of each gene and the bias of the nucleotide composition. The AT-skew and GC-skew were calculated as follows: AT-skew = (A − T)/(A + T), and GC-skew = (G − C)/(G + C). The relative synonymous codon usage (RSCU) of the newly sequenced species was performed by MEGA X. Mitogenome maps of each newly sequenced species were generated by an online server CGview (https://cgview.ca/, accessed on 30 April 2025).

### 2.3. Phylogenetic Analysis

To reconstruct the phylogenetic relationships of Halictidae, we selected 10 samples, including eight ingroups and two Colletidae species as outgroups based on prior research of bees [32]. Thirteen PCGs and two rRNA genes of all 10 species were used for phylogenetic analyses. The nucleotide and protein sequences of all samples were aligned by MAFFT v7.450 (Osaka, Japan) with the L-INS-I method [33]. Sequence trimming was conducted via Trimal v1.4.1 (Barcelona, Spain) [34] with “-automated1” strategy. Finally, five matrices were generated to reconstruct the phylogenetic relationship, and FASconCAT-G v1.04 (Santa Cruz, CA, USA) [35] was performed to concatenate each matrix: (1) cds_faa matrix, including all PCG amino acid reads; (2) cds_fna matrix, containing all PCG nucleotide reads; (3) cds_rrna matrix, including all PCG and two rRNA nucleotide reads; (4) cds12_fna matrix, containing all PCG nucleotide reads except the third codon positions; (5) cds12_rrna matrix, including all PCG nucleotide reads, which removed the third codon positions and two rRNA genes.

For all matrices, we used ML and BI methods to reconstruct the phylogenetic relationship within Halictidae. For ML analyses, we used ModelFinder [36] in IQ-TREE 2 (Canberra, ACT, Australia) [37] to select the best substitution model for each matrix. In addition, we used the posterior mean site frequency (PMSF) model [38] (‘−m − mtART + C60 + FO + R’) to reconstruct the phylogenetic relationship of Halictidae in IQ-TREE for matric cds_faa to minimize long-branch attraction artifacts. Phylobayes-MPI (Montréal, QC, Canada) [39] was applied to generate the BI tree using the site-heterogeneous mixture model (−m CAT + GTR). We performed two independent Markov Chain Monte Carlo (MCMC) chains, each with 10,000,000 generations, stopping once we achieved satisfactory convergence (maxdiff < 0.3). Finally, iTOL was used to beautify the phylogenetic tree (https://itol.embl.de/upload.cgi, accessed on 20 April 2025).

## 3. Results

### 3.1. Mitogenomic Organization

**Figure 1:**
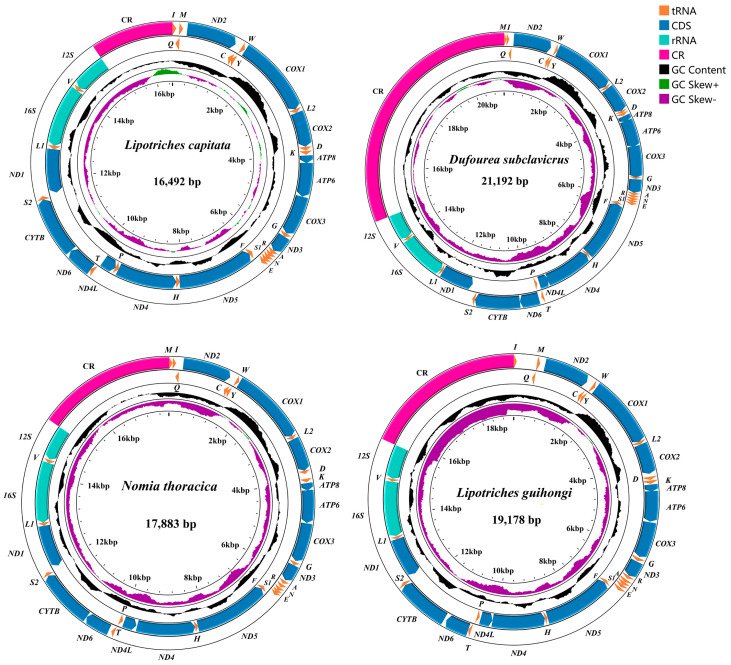
The mitogenome map of the newly sequenced species. The arrow indicates the direction of gene transcription. We used normative abbreviations to represent PCGs and rRNAs, and single-letter abbreviations were used to represent tRNAs. GC content of the complete mitogenome is shown in the second circle. GC-skew of the complete mitogenome is shown in the third circle. The innermost circle shows the length of the complete third item.

We sequenced about 6 Gb of raw data for each species. Four species (*Nomia thoracica*, *Lipotriches guihongi*, *Lipotriches capitata*, *Dufoure subclavicrus*) of Halictidae were obtained, all of which were linear mitogenomes. All newly obtained mitogenomes have been submitted to GenBank under accession numbers PQ050612–PQ050615 (Table 1). For newly obtained mitogenomes, the whole length ranged from 16,492 (*L. capitata*) to 21,192 bp (*D. subclavicrus*, Table 2). We also identified one CR, two rRNAs, 22 tRNAs, and 13 PCGs (Figure 1). All newly sequenced mitogenomes showed positive AT skew, except *L. capitata*, ranging from −0.031 (*L. capitata*) to 0.047 (*D. subclavicrus*). The GC skew for all newly reported mitogenomes was negative, ranging from −0.364 (*L. guihongi*) to −0.112 (*L. capitata*, Table 2). The GC content (%) ranged from 13.55 (*L. capitata*) to 17.38 (*D. subclavicrus*), and the AT content (%) ranged from 82.55 (*D. subclavicrus*) to 86.41 (*L. capitata*, Table 2).

### 3.2. Protein-Coding Genes, Codon Usage, and Evolutionary Rates

The sizes of PCGs, tRNAs, and rRNAs of newly sequenced species are similar. The PCG lengths of the newly obtained species ranged from 11,075 (*D. subclavicrus*) to 11,142 (*L. guihongi*). PCGs of all newly obtained mitogenomes showed negative AT skew, ranging from −0.122 (*L. capitata*) to −0.069 (*D. subclavicrus*). The GC skew for PCGs of all newly reported mitogenomes were negative, except *L. capitata*, ranging from −0.153 (*D. subclavicrus*) to 0.026 (*L. capitata*, Table 2). The GC content (%) of PCGs ranged from 13.39 (*L. capitata*) to 18.18 (*D. subclavicrus*), and the AT content (%) ranged from 81.82 (*D*. *subclavicrus*) to 86.61 (*L. capitata*, Table 2).

CR sizes of *L. capitata* were the shortest (1576 bp), while *D. subclavicrus* exhibited the longest CR sizes (6572 bp). All newly sequenced and assembled mitogenomes were started with ATN. However, varying start codons were found: *ATP6* and *CYTB* used ATG as the start codon in two species; *COX1*, *COX2*, *ND1*, *ND2*, *ND5,* and *ND6* used ATA as the start codon in one species; *ATP8*, *COX2*, *ND3*, *ND4*, *ND6,* and COX1 used ATT as the start codon in two species; *ND4L*, *COX1*, and *COX2* used ATC as the start codon in one species; *COX1* used TTG as the start codon in one species and so on (Figure S1). TAA or TAG are the common stop codons of this group, whereas *COX1*, *ND4*, and *ND5* in one species have TA or T as the stop codons, which were incomplete termination codons (Figure S2). The termination codons of PCGs are frequently found to be incomplete in insects, and they are commonly completed through a process known as polyadenylation, which occurs after the excision of the downstream tRNA gene [40,41,42,43].

Twenty-two tRNAs were identified, ranging from 50 to 77 bp in length. The tRNA characters of newly obtained mitogenomes are as follows: AT content (%) ranged from 84.11 (*D. subclavicrus*) to 88.20 (*L. capitata*); the GC content (%) ranged from 11.80 (*L. capitata*) to 15.89 (*D. subclavicrus*); and the AT skew and GC skew exhibited positive values (Table 2).

**Figure 2:**
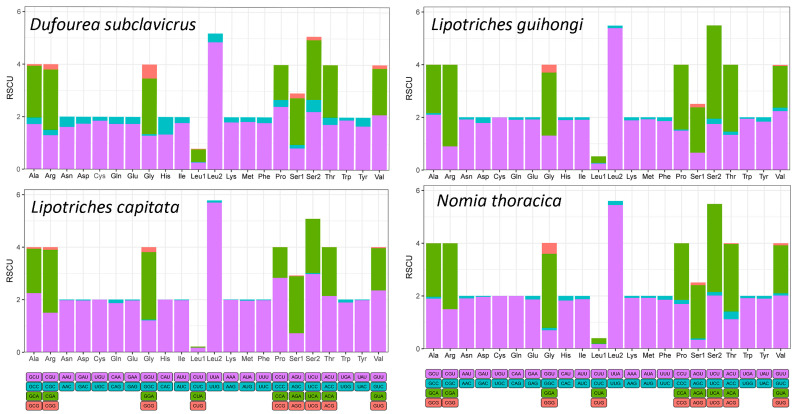
Relative synonymous codon usage (RSCU) of mitochondrial protein-coding genes of newly sequenced species. The *X*-axis shows different amino acids, and the *Y*-axis shows the RSCU value (the number of times a certain synonymous codon is used/the average number of times that all codons coding the amino acid are used).

**Figure 3:**
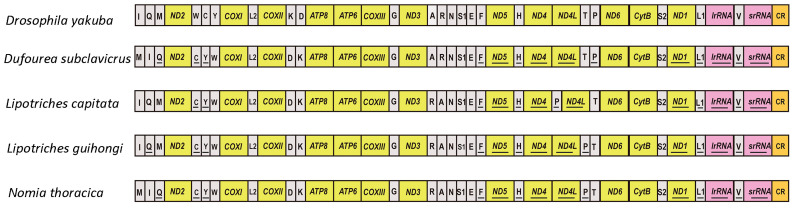
Gene arrangements of newly sequenced mitochondrial genomes. The mitochondrial genome is color-coded as follows: white corresponds to protein-coding regions, green designates tRNA genes, and red marks structural rearrangement sites.

Four newly sequenced mitochondrial genomes exhibited congruent relative synonymous codon usage (RSCU) patterns (Figure 2), which showed the RSCU values for all synonymous codons corresponding to the 22 amino acids, utilizing the 62 available codons in the 13 PCGs of Nomiinae species. Compared to the newly reported species, we found that the preferred codons were UUU, UUA, and AUU. Meanwhile, the frequently utilized amino acids were Leu2, Phe, and IIe, indicating that A/T was the preferred nucleotide composition. The codons with RSCU > 2 values for most newly sequenced species were as follows: UUA > UCA > CGA, while for *L. capitata,* they were UUA > UCU > GGA (Figure 3, Tables S1–S4). For amino acid, NNA and NNU were the most frequently used codons, reflecting the AT preference in nucleotide composition (Tables S1–S4).

### 3.3. Gene Rearrangement

In this study, we used the gene arrangements of *Drosophila yakuba* as the ancestral state [44]. Gene rearrangements existed in all newly sequenced species, and five gene rearrangement patterns were found in total (Figure 3). The gene cluster *trnI-trnQ-trnM* was frequently rearranged to *trnM-trnI-trnQ* in three species (*D. subclavicrus*, *L. capitata*, *N. thoracica*, Figure 3); the *trnK-trnD* tRNA block was rearranged in all newly sequenced species (*COX2-trnK-trnD* to *COX2-trnD-trnK*); the *trnR* was inverted in all newly sequenced Nomiinae species (*ND3-trnA-trnR-trnN* transformed into *ND3-trnR-trnA-trnN*); *trnP* was inverted in *L. capitata* and *N. thoracica* (*ND4-ND4L-trnT-trnP* to *ND4 -ND4L-trnP-trnT*, Figure 3). The *ND4-ND4L-trnT-trnP* gene cluster was rearranged to *ND4-trnP-ND4L-trnT* in *L. guihongi* (Figure 3), which was the first instance of mitochondrial gene rearrangement found in Apoidea.

### 3.4. Phylogenetic Relationships

**Figure 4:**
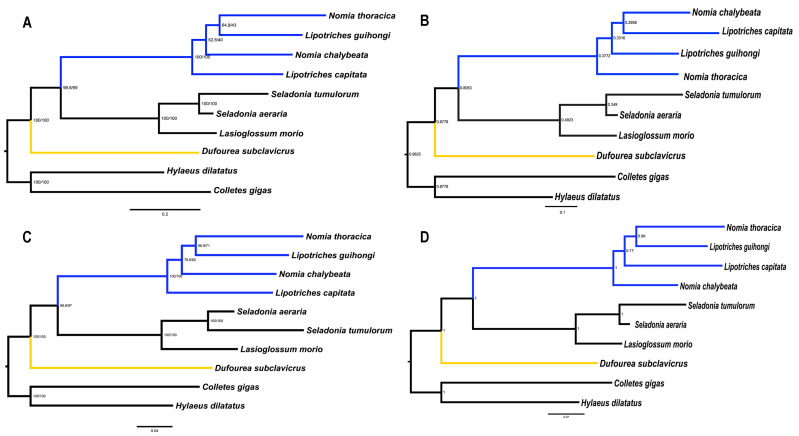
Phylogenetic trees of Halictidae: (**A**) ML tree based on analysis cds_faa with PMSF model in IQTREE. (**B**) BI tree based on analysis cds_fna in Phylobayes. (**C**) ML tree based on the analysis cds12_rrna with partition model in IQTREE. (**D**) BI tree based on the analysis cds12_rrna in Phylobayes. Support values on nodes indicate Bayesian posterior probabilities in topology (**B**,**D**), while they represent SH-aLRT/UFBoot2 in topology (**A**,**C**).

Five matrices were used to reconstruct the phylogenetic relationships of the Halictidae: cds_faa (5375 sites), cds_fna (7148 sites), cds_rrna (12,580 sites), cds12_fna (7148 sites), and cds12_rrna (9006 sites). We used five matrices to generate four phylogenetic topologies based on BI and ML approaches (Figure 4 and Figures S3–S9). These results showed that all of them supporting Rophitinae were the basal group within the Halictidae (Figure 4 and Figures S3–S9). For the Nomiinae, there were four different topologies, all of which show that *Austronomia* is not a subgenus within the genus *Lipotriches*, and the subgenus *Gnathonomia* may be treated as a genus within Nomiinae. Topology 1 (T1) was generated from cds_faa, cds_rrna using BI methods, cds_faa, cds_fna, cds_rrna using the partition model, and cds_faa using the PMSF model: (((*Gnathonomia*, *Lipotriches*), *Nomia*), *Austronomia*) (Figure 4A, Figures S3, S4 and S7–S9). This topology suggested that the subgenus *Gnathonomia* is relatively close to the genus *Lipotriches*. Topology 2 (T2) was generated using cds_fna, cds12_fna based on the BI method, and cds12_fna based on the partition model: ((*Nomia*, *Austronomia*), *Lipotriches*, *Gnathonomia*) (Figure 4B, Figures S5 and S6). Here, the genus *Nomia* is close to *Austronomia*, and the *Gnathonomia* is the basal lineage. Topology 3 (T3) was generated using the matric cds12_rrna based on the partition model: (((*Gnathonomia*, *Lipotriches*), *Austronomia*), *Nomia*) (Figure 4C). In T3, the subgenus *Gnathonomia* is close to the genus *Lipotriches*. Topology 4 (T4) was generated by the matric cds12_rrna based on BI methods: (((*Gnathonomia*, *Lipotriches*), *Nomia*), *Austronomia*) (Figure 4D). This topology also supported that the subgenus *Gnathonomia* is close to the genus *Lipotriches*. Overall, the phylogenetic relationships of the subfamily Nomiinae were highly unstable with different models.

## 4. Discussion

### 4.1. Mitochondrial Genome Structure

We newly sequenced and assembled four mitochondrial genomes within Halictidae. All newly obtained mitochondrial genomes had typical circular double-stranded structures, and the genes exhibited high conservation in length. The arrangement order and nucleotide composition of newly reported data are similar to those of other published insect mitochondrial genomes [18,19,22,23,24]. The length of the mitogenomes was significantly different among species (Figure 2, Table 2), mainly because of variations in control region size. The nucleotide composition of newly sequenced species exhibited a considerable bias toward A + T, and the A + T content was higher than 80% in all species (81.82–86.61%, Table 2), which was commonly observed in published Hymenoptera mitochondrial genomes [45,46]. There were no remarkable differences in base composition discovered among the newly sequenced mitochondrial genomes (Table 2). For insects, AT skew in mitogenomes exhibits positive values in general, while our results showed a negative AT skew for *L capitata*, and all GC skew values were negative (Table 2), indicating a preference for thymine (T) and guanine (G) bases in Hymenoptera, and similar results have been published for other Hymenoptera and bee species [45,46].

### 4.2. Mitogenomic Gene Rearrangements

Gene rearrangement in insect mitochondrial genomes was uncommon but has been reported in Thysanoptera, Psocodea, Hemiptera, Hymenoptera, and Trichoptera [47,48,49,50,51]. To date, four main types of gene rearrangements have been observed in insects: local inversion, remote inversion, gene shuffling, and translocation [52]. In Apoidea, inversion and inverse transposition events occurred at a relatively high frequency, similarly to observations in other hymenopterans [50,53,54]. Indeed, insects in the order Hymenoptera are known to exhibit an exceptionally high level of mitochondrial gene rearrangement, which often carries important phylogenetic signals [17,46,47,51]. However, our understanding of the mechanism underlying gene rearrangement within Halictidae remains limited, largely due to the limited samples.

Contrasted with the putative ancestral gene rearrangement of insects, all four newly sequenced mitochondrial genomes presented gene rearrangement (Figure 3). We found five gene rearrangements in the newly sequenced species, and all rearranged genes were tRNA genes (Figure 3). In this study, *COX2-trnD-trnK* and *ND3-trnR-trnA-trnN* were observed in all newly sequenced species. *trnM-trnI-trnQ* was also found in most species of our study, except *L. guihongi.* Gene shuffling (*trnQ/trnM*, *trnW/trnC-trnY*, and *trnK/trnD*) was found to be a significant rearrangement pattern in bees, which had been proposed as an important type of gene rearrangement in Hymenoptera [52,53,54]. The gene rearrangement *CR-trnI-trnQ-trnM-nad2-trnW-trnC-trnY* was the main hot spot in bees, and our study showed that the gene rearrangement hot spot of Halictidae also exists in this region (Figure 3). In addition, this study described a special rearrangement of the *ND4-trnP-ND4L-trnT* in *L. guihongi* (Figure 3), which was the first instance of mitochondrial gene rearrangement found in bees, providing a foundation for research on Halictidae biological characteristics. In theory, 37 genes of the mitochondrial genome have considerable potential for rearrangement. Therefore, different species are unlikely to adopt the same gene sequence. The shared gene sequence is more likely to indicate a common evolutionary history [17,46,47]. Most gene rearrangement patters in this study were observed in all newly sequenced species, showing that those gene rearrangements in tRNAs may be a common feature within the Halictidae.

### 4.3. Phylogenetic Relationship Analyses

Herein, we used mitochondrial genomes to reconstruct the relationships within Halictidae. Five matrices based on BI and ML approaches yielded four different topologies; all topologies supported that the Rophitinae was the basal linage of Halictidae (Figure 4 and Figures S3–S9), which was similar to the results of Peskon and Danforth [55,56,57]. The former used morphological characteristics, while the latter used nuclear genes to reconstruct the phylogenetic relationships within Halictidae 15 years ago [55,56,57].

Halictidae is an important bee family, not only because its members contribute to pollination, but also because of their social behavior [4]. However, there have been relatively few studies on the phylogeny of this group in recent years, especially for Nomiinae. Previous studies of Nomiinae mainly focused on morphology. The molecular data are seriously inadequate, and the relationships in this group have led to great controversy based on morphological characters. Michener divided Nomiinae into 11 genera and 19 subgenera, while Pauly treated almost all subgenera as independent genera [3,4,10,58,59]. Pauly’s classification perspectives have not been widely supported by other taxonomists. In this study, all topologies showed that *Austronomia* was not a subgenus within the genus *Lipotriches*, and *Gnathonomia* may also be treated as a genus rather than as a subgenus of *Nomia* (Figure 4 and Figures S3–S9), which closely resembles the generic system of Pauly [5]. Unfortunately, due to limited samples and phylogenetic signal markers, we have not yet resolved the phylogenetic relationships of Nomiinae. In the future, more samples of genera and subgenera will be included to explore the phylogenetic relationships within this group, and we will use more efficient data, including low-coverage whole-genome genome and ultra-conserved element data to resolve the relationships within Nomiinae, which show great promise in phylogenetic research [60,61,62].

## 5. Conclusions

In this study, we newly sequenced, assembled, and annotated four mitogenomes of Halictidae. Compared to previously published data, all newly obtained mitogenomes had similar structural characters and nucleotide compositions. In addition, gene rearrangement analysis found one novel model: *ND4-trnP-ND4L-trnT*. Thus, our study provided more available mitogenomes for the study of Nomiinae and Halictidae. In adding two Halictinae species, we reconstructed the phylogenetic relationships within Halictidae based on BI and ML methods. All topologies showed that Rophitinae is the basal group within Halictidae. Our research provides a molecular database for research on Halictidae. Further study, including the addition of more samples and using more phylogenetic signal markers (e.g. UCEs, whole-genome sequencing), are needed to define the phylogenetic relationships within the subfamily and the evolutionary biology of Halictidae.

## Figures and Tables

**Table 1:** Detailed information on newly sequenced species in this study.

Species	Location	Longitude and Latitude	Elevation (m)	Date	Collector	Accession Number
*Nomia thoracica*	Hainan, Sanya	16.745° E, 22.111° N	50	28 June 2017	Feng Yuan	PQ050612
*Lipotriches guihongi*	Xizang, Jilong	28.226° N, 85.214° E	2713	20 July 2018	Dan Zhang	PQ050613
*Lipotriches capitata*	Yunnan, Xishuangbanna	22.401° N, 100.235° E	1240	16 May 2014	Dan Zhang	PQ050614
*Dufourea subclavicrus*	Xizang, Yadong	27.246° N, 88.515° E	3995	6 July 2018	Dan Zhang	PQ050615

**Table 2:** Nucleotide composition of newly sequenced species.

Species	Regions	Length (bp)	a	t	c	g	AT	GC	AT-Skew	GC-Skew
*Nomia thoracica*	Whole genome	17,883	44.03	41.36	9.32	5.23	85.39	14.56	0.031	−0.281
	PCGs	11,114	38.44	46.32	8.25	6.99	84.76	15.24	−0.093	−0.083
	Site 1	3705	41.69	40.46	7.68	10.17	82.15	17.85	0.015	0.140
	Site 2	3705	25.41	51.50	13.55	9.54	76.91	23.09	−0.339	−0.174
	Site 3	3704	48.23	46.99	3.53	1.25	95.22	4.78	0.013	−0.477
	tRNA	1455	45.36	41.58	5.57	7.49	86.94	13.06	0.043	0.147
	rRNA	2097	42.87	43.44	4.49	9.14	86.31	13.62	−0.007	0.341
	CR	2755	44.07	44.39	7.33	4.07	88.46	11.40	−0.004	−0.287
*Lipotriches guihongi*	Whole genome	19,178	43.68	42.76	9.24	4.31	86.44	13.56	0.011	−0.364
	PCGs	11,142	39.31	45.71	8.23	6.76	85.01	14.99	−0.075	−0.098
	Site 1	3714	43.81	38.20	8.21	9.78	82.02	17.98	0.068	0.087
	Site 2	3714	24.53	52.75	13.36	9.37	77.28	22.72	−0.365	−0.176
	Site 3	3714	49.57	46.17	3.13	1.13	95.74	4.26	0.035	−0.470
	tRNA	1523	45.24	42.48	5.25	7.03	87.72	12.28	0.031	0.144
	rRNA	2160	43.26	43.95	4.27	8.53	87.20	12.80	−0.008	0.333
	CR	3481	42.23	49.27	8.07	0.40	91.50	8.47	−0.077	−0.905
*Lipotriches capitata*	Whole genome	16,492	41.84	44.56	7.54	6.02	86.41	13.55	−0.031	−0.112
	PCGs	11,127	38.02	48.59	6.52	6.87	86.61	13.39	−0.122	0.026
	Site 1	3710	42.46	40.43	7.19	9.91	82.90	17.10	0.024	0.159
	Site 2	3709	25.48	52.93	12.10	9.48	78.41	21.59	−0.350	−0.121
	Site 3	3708	46.12	52.39	0.28	1.21	98.51	1.49	−0.064	0.620
	tRNA	1492	44.24	43.97	4.56	7.24	88.20	11.80	0.003	0.227
	rRNA	2116	44.13	43.53	4.26	8.08	87.67	12.34	0.007	0.310
	CR	1576	43.46	43.72	6.79	5.58	87.18	12.37	−0.003	−0.097
*Dufourea subclavicrus*	Whole genome	21,192	43.23	39.32	11.04	6.35	82.55	17.38	0.047	−0.270
	PCGs	11,075	38.09	43.73	10.48	7.70	81.82	18.18	−0.069	−0.153
	Site 1	3692	44.45	36.15	9.22	10.19	80.59	19.41	0.103	0.050
	Site 2	3692	24.21	51.60	14.39	9.80	75.81	24.19	−0.361	−0.190
	Site 3	3691	45.63	43.44	7.82	3.12	89.07	10.93	0.025	−0.430
	tRNA	1441	42.61	41.50	6.94	8.95	84.11	15.89	0.013	0.127
	rRNA	2047	40.72	42.92	5.65	10.73	83.63	16.37	−0.026	0.310
	CR	6572	40.73	43.75	8.31	6.98	84.48	15.29	−0.036	−0.087

## Data Availability

The data are contained within the article or Supplementary Materials.

## Supplementary Materials

The following supporting information can be downloaded at: https://www.mdpi.com/article/10.3390/ani15152234/s1. Table S1. Relative synonymous codon usages (RSCUs) of PCGs of *Nomia thoracica.* Table S2. Relative synonymous codon usages (RSCUs) of PCGs of *Lipotriches guihongi*. Table S3. Relative synonymous codon usages (RSCUs) of PCGs of *Lipotriches capitata*. Table S4. Relative synonymous codon usages (RSCUs) of PCGs of *Dufourea subclavicrus.* Figure S1. Start codons of protein-coding genes among newly obtained mitogenomes. Figure S2. Stop codons of protein-coding genes of newly obtained mitogenomes. Figure S3. Maximum likelihood phylogenetic tree of Halictidae based on the analysis cds_faa with a partitioned model in IQTREE. Support values on nodes indicate SH-aLRT/UFBoot2, respectively. Figure S4. Bayesian inference phylogenetic tree of Halictidae based on the analysis cd_faa with a GTR + CAT model in phylobayes. Support values on nodes indicate Bayesian posterior probabilities. Figure S5. Bayesian inference phylogenetic tree of Halictidae based on the analysis cds12_fna with a GTR + CAT model in phylobayes. Support values on nodes indicate Bayesian posterior probabilities. Figure S6. Maximum likelihood phylogenetic tree of Halictidae based on the analysis cds12_fna with a partitioned model in IQTREE. Support values on nodes indicate SH-aLRT/UFBoot2, respectively. Figure S7. Bayesian inference phylogenetic tree of Halictidae based on the analysis cds_rrna with a GTR + CAT model in phylobayes. Support values on nodes indicate Bayesian posterior probabilities. Figure S8. Maximum likelihood phylogenetic tree of Halictidae based on the analysis cds_rrna with a partitioned model in IQTREE. Support values on nodes indicate SH-aLRT/UFBoot2, respectively. Figure S9. Maximum likelihood phylogenetic tree of Halictidae based on the analysis cds_fna with a partitioned model in IQTREE. Support values on nodes indicate SH-aLRT/UFBoot2, respectively.
