# Supplementary material for: Comparative Analyses Reveal Mitogenome Characteristics of Halictidae and Novel Rearrangement (Hymenoptera: Apoidea: Anthophila)
Source: Animals (Basel). 2025 Jul 30;15(15):2234. doi: 10.3390/ani15152234 (PMC12345510; doi:10.3390/ani15152234)
Supplement: Supplementary file 1 [file animals-15-02234-s001.zip › animals-3699316-supplementary.pdf]

Table S1 Relative synonymous codon usages (RSCUs) of PCGs of *Nomia thoracica*

**Codon**	**Count**	**RSCU**	**Codon**	**Count**	**RSCU**
UUU	406.0	1.86	UCU	78.0	2.02
UUC	30.0	0.14	UCC	5.0	0.13
UUA	498.0	5.45	UCA	129.0	3.34
UUG	14.0	0.15	UCG	0.0	0.0
CUU	16.0	0.18	CCU	45.0	1.70
CUC	1.0	0.01	CCC	4.0	0.15
CUA	19.0	0.21	CCA	57.0	2.15
CUG	0.0	0.00	CCG	0.0	0.00
AUU	433.0	1.88	ACU	38.0	1.12
AUC	28.0	0.12	ACC	10.0	0.29
AUA	339.0	1.93	ACA	87.0	2.56
AUG	12.0	0.07	ACG	1.0	0.03
GUU	51.0	2.02	GCU	29.0	1.90
GUC	2.0	0.08	GCC	1.0	0.07
GUA	46.0	1.82	GCA	31.0	2.03
GUG	2.0	0.08	GCG	0.0	0.00
UAU	203.0	1.90	UGU	32.0	2.00
UAC	11.0	0.10	UGC	0.0	0.00
UAA*	0.0	0.00	UGA	73.0	1.92
UAG*	0.0	0.00	UGG	3.0	0.08
CAU	55.0	1.83	CGU	15.0	1.50
CAC	5.0	0.17	CGC	0.0	0.00
CAA	43.0	2.00	CGA	25.0	2.50
CAG	0.0	0.00	CGG	0.0	0.00
AAU	263.0	1.91	AGU	13.0	0.34
AAC	13.0	0.09	AGC	2.0	0.05
AAA	163.0	1.93	AGA	78.0	2.02
AAG	6.0	0.07	AGG	4.0	0.10
GAU	59.0	1.97	GGU	24.0	0.70
GAC	1.0	0.03	GGC	3.0	0.09
GAA	70.0	1.87	GGA	97.0	2.81
GAG	5.0	0.13	GGG	14.0	0.41

* Stop codon

Table S2 Relative synonymous codon usages (RSCUs) of PCGs of *Lipotriches guihongi*

**Codon**	**Count**	**RSCU**	**Codon**	**Count**	**RSCU**
UUU	375.0	1.86	UCU	69.0	1.75
UUC	28.0	0.14	UCC	8.0	0.20
UUA	499.0	5.39	UCA	140.0	3.54
UUG	8.0	0.09	UCG	0.0	0.00
CUU	23.0	0.25	CCU	42.0	1.50
CUC	2.0	0.02	CCC	1.0	0.04
CUA	23.0	0.25	CCA	69.0	2.46
CUG	0.0	0.00	CCG	0.0	0.00
AUU	464.0	1.91	ACU	46.0	1.34
AUC	22.0	0.09	ACC	4.0	0.12
AUA	362.0	1.93	ACA	87.0	2.54
AUG	13.0	0.07	ACG	0.0	0.00
GUU	51.0	2.24	GCU	33.0	2.10
GUC	3.0	0.13	GCC	1.0	0.06
GUA	36.0	1.58	GCA	29.0	1.84
GUG	1.0	0.04	GCG	0.0	0.00
UAU	200.0	1.84	UGU	29.0	2.00
UAC	17.0	0.16	UGC	0.0	0.00
UAA*	0.0	0.00	UGA	75.0	1.95
UAG*	0.0	0.00	UGG	2.0	0.05
CAU	56.0	1.90	CGU	9.0	0.90
CAC	3.0	0.10	CGC	0.0	0.00
CAA	45.0	1.91	CGA	31.0	3.10
CAG	2.0	0.09	CGG	0.0	0.00
AAU	262.0	1.92	AGU	26.0	0.66
AAC	11.0	0.08	AGC	0.0	0.00
AAA	150.0	1.89	AGA	68.0	1.72
AAG	9.0	0.11	AGG	5.0	0.13
GAU	51.0	1.79	GGU	44.0	1.31
GAC	6.0	0.21	GGC	0.0	0.00
GAA	68.0	1.92	GGA	80.0	2.39
GAG	3.0	0.08	GGG	10.0	0.30

* Stop codon

Table S3 Relative synonymous codon usages (RSCUs) of PCGs of *Lipotriches capitata*

Codon	Count	RSCU	Codon	Count	RSCU
UUU	389.0	1.98	UCU	115.0	2.98
UUC	4.0	0.02	UCC	1.0	0.03
UUA	559.0	5.70	UCA	80.0	2.07
UUG	8.0	0.08	UCG	0.0	0.00
CUU	17.0	0.17	CCU	73.0	2.83
CUC	0.0	0.00	CCC	0.0	0.00
CUA	4.0	0.04	CCA	30.0	1.17
CUG	0.0	0.00	CCG	0.0	0.00
AUU	474.0	1.98	ACU	61.0	2.14
AUC	4.0	0.02	ACC	0.0	0.00
AUA	377.0	1.96	ACA	53.0	1.86
AUG	7.0	0.04	ACG	0.0	0.00
GUU	54.0	2.35	GCU	36.0	2.25
GUC	0.0	0.00	GCC	0.0	0.00
GUA	37.0	1.61	GCA	27.0	1.69
GUG	1.0	0.04	GCG	1.0	0.06
UAU	230.0	1.98	UGU	29.0	2.00
UAC	2.0	0.02	UGC	0.0	0.00
UAA*	0.0	0.00	UGA	71.0	1.89
UAG*	0.0	0.00	UGG	4.0	0.11
CAU	58.0	2.00	CGU	15.0	1.50
CAC	0.0	0.00	CGC	0.0	0.00
CAA	43.0	1.87	CGA	24.0	2.40
CAG	3.0	0.13	CGG	1.0	0.10
AAU	263.0	1.98	AGU	28.0	0.72
AAC	2.0	0.02	AGC	0.0	0.00
AAA	161.0	1.99	AGA	84.0	2.17
AAG	1.0	0.01	AGG	1.0	0.03
GAU	62.0	1.97	GGU	38.0	1.21
GAC	1.0	0.03	GGC	1.0	0.03
GAA	75.0	1.97	GGA	81.0	2.57
GAG	1.0	0.03	GGG	6.0	0.19

* stop codon

Table S4 Relative synonymous codon usages (RSCUs) of PCGs of *Dufourea subclavicrus*

**Codon**	**Count**	**RSCU**	**Codon**	**Count**	**RSCU**
UUU	375.0	1.86	UCU	69.0	1.75
UUC	28.0	0.14	UCC	8.0	0.20
UUA	499.0	5.39	UCA	140.0	3.54
UUG	8.0	0.09	UCG	0.0	0.00
CUU	23.0	0.25	CCU	42.0	1.50
CUC	2.0	0.02	CCC	1.0	0.04
CUA	23.0	0.25	CCA	69.0	2.46
CUG	0.0	0.00	CCG	0.0	0.00
AUU	464.0	1.91	ACU	46.0	1.34
AUC	22.0	0.09	ACC	4.0	0.12
AUA	362.0	1.93	ACA	87.0	2.54
AUG	13.0	0.07	ACG	0.0	0.00
GUU	51.0	2.24	GCU	33.0	2.10
GUC	3.0	0.13	GCC	1.0	0.06
GUA	36.0	1.58	GCA	29.0	1.84
GUG	1.0	0.04	GCG	0.0	0.00
UAU	200.0	1.84	UGU	29.0	2.00
UAC	17.0	0.16	UGC	0.0	0.00
UAA*	0.0	0.00	UGA	75.0	1.95
UAG*	0.0	0.00	UGG	2.0	0.05
CAU	56.0	1.90	CGU	9.0	0.90
CAC	3.0	0.10	CGC	0.0	0.00
CAA	45.0	1.91	CGA	31.0	3.10
CAG	2.0	0.09	CGG	0.0	0.00
AAU	262.0	1.92	AGU	26.0	0.66
AAC	11.0	0.08	AGC	0.0	0.00
AAA	150.0	1.89	AGA	68.0	1.72
AAG	9.0	0.11	AGG	5.0	0.13
GAU	51.0	1.79	GGU	44.0	1.31
GAC	6.0	0.21	GGC	0.0	0.00
GAA	68.0	1.92	GGA	80.0	2.39
GAG	3.0	0.08	GGG	10.0	0.30

* stop codon

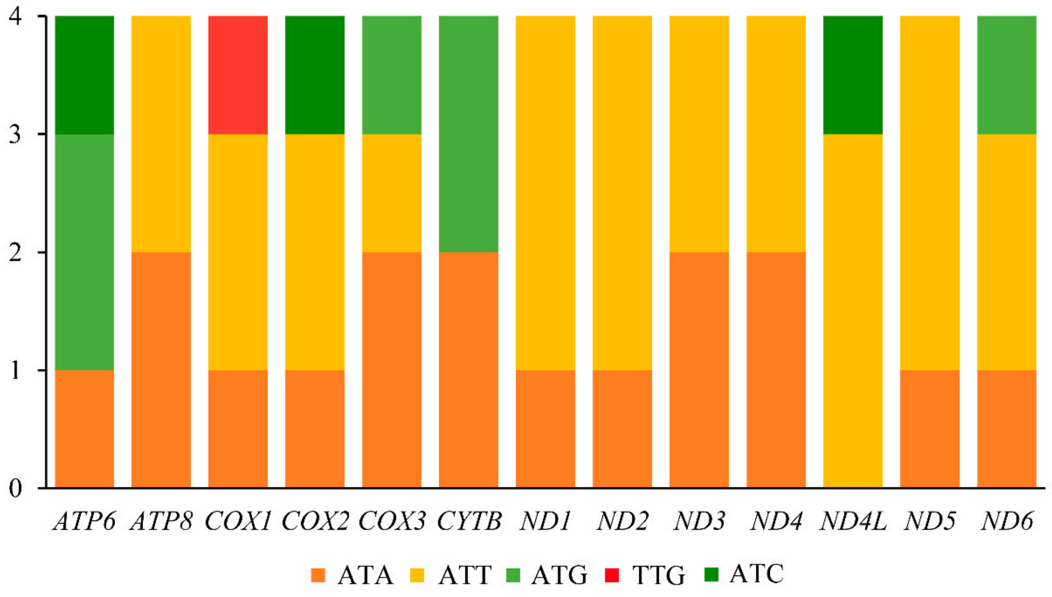

Figure S1. Start codons of protein-coding genes among newly obtained mitogenomes

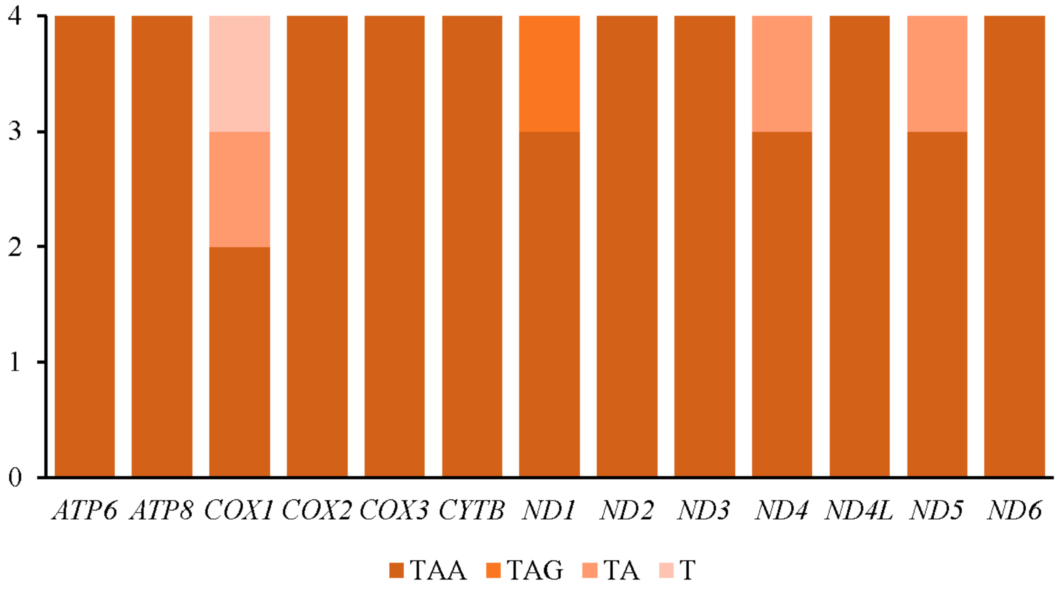

Figure S2. Stop codon of protein-coding genes among newly obtained mitogenomes

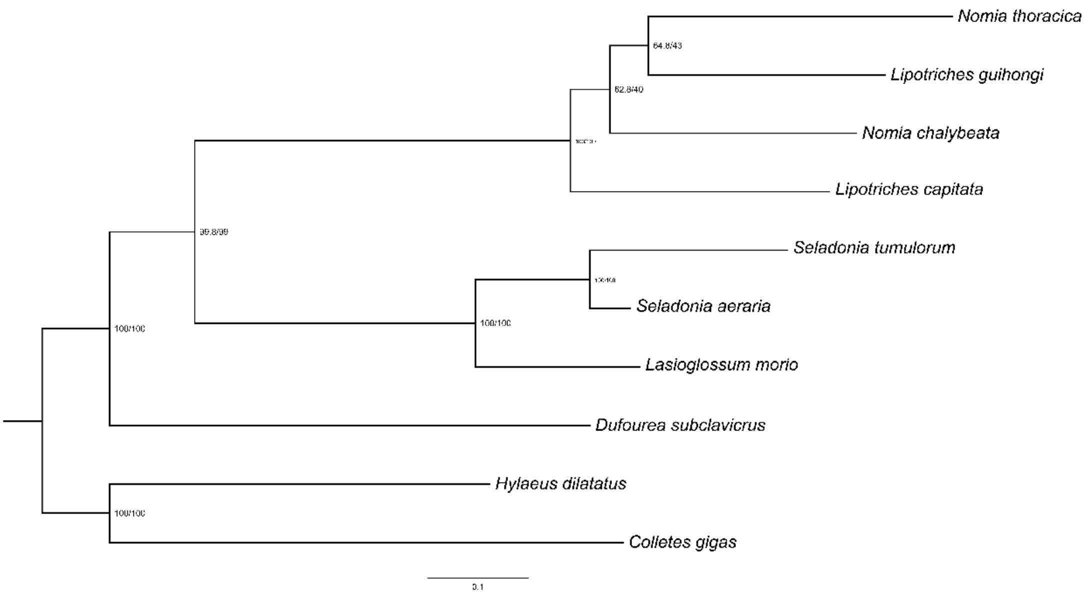

Figure S3 Maximum likelihood phylogenetic tree of Halictidea based on the analysis cds_faa with a Partitioned model in IQTREE. Support values on nodes indicate SH-aLRT/UFBoot2, respectively.

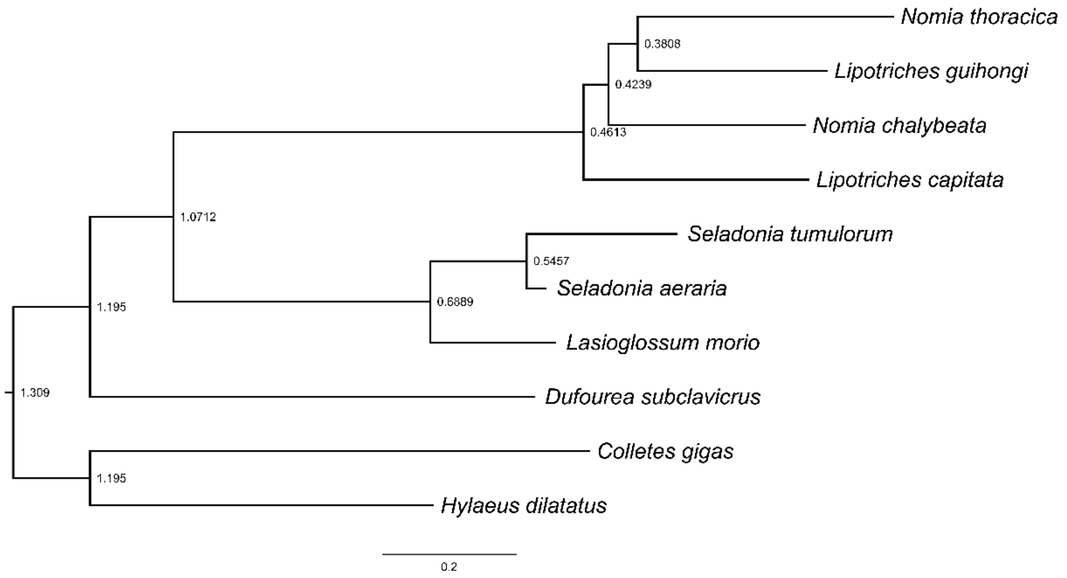

Figure S4. Bayesian inference phylogenetic tree of Halictidae based on the analysis cd_faa with a GTR + CAT model in phylobayes. Support values on nodes indicate Bayesian posterior probabilities.

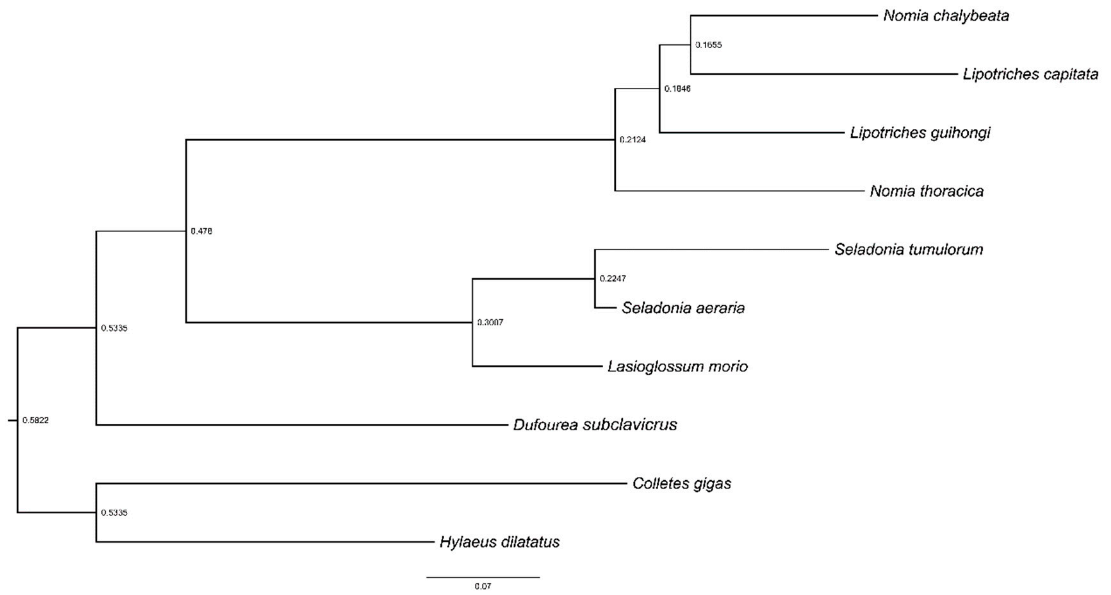

Figure S5. Bayesian inference phylogenetic tree of Halictidae based on the analysis cds12_fna with a GTR + CAT model in phylobayes. Support values on nodes indicate Bayesian posterior probabilities.

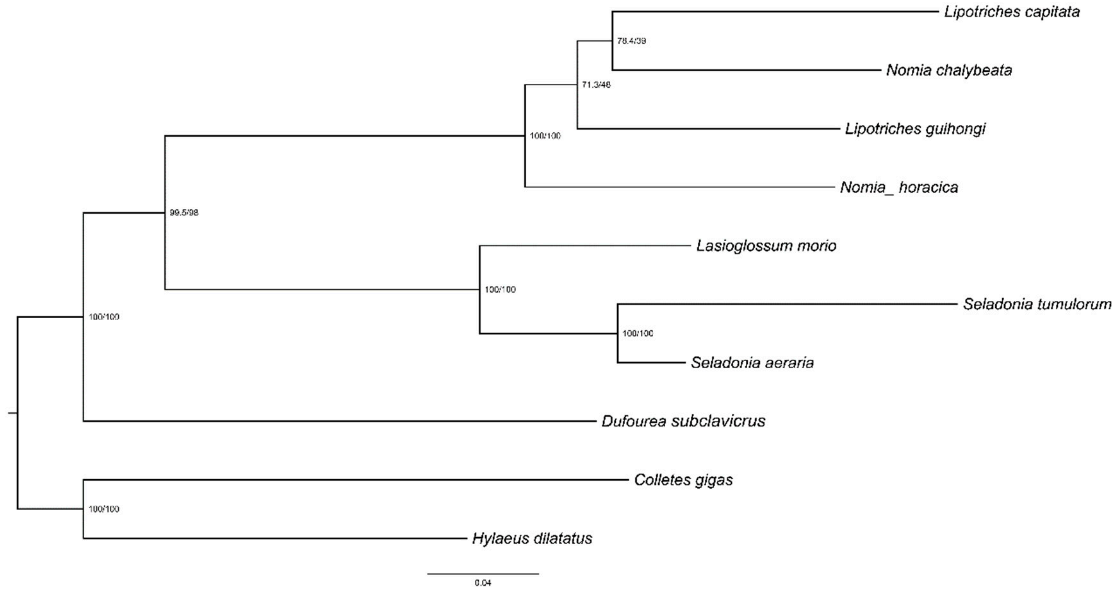

Figure S6. Maximum likelihood phylogenetic tree of Halictidae based on the analysis cds12_fna with a Partitioned model in IQTREE. Support values on nodes indicate SH-aLRT/UFBoot2, respectively.

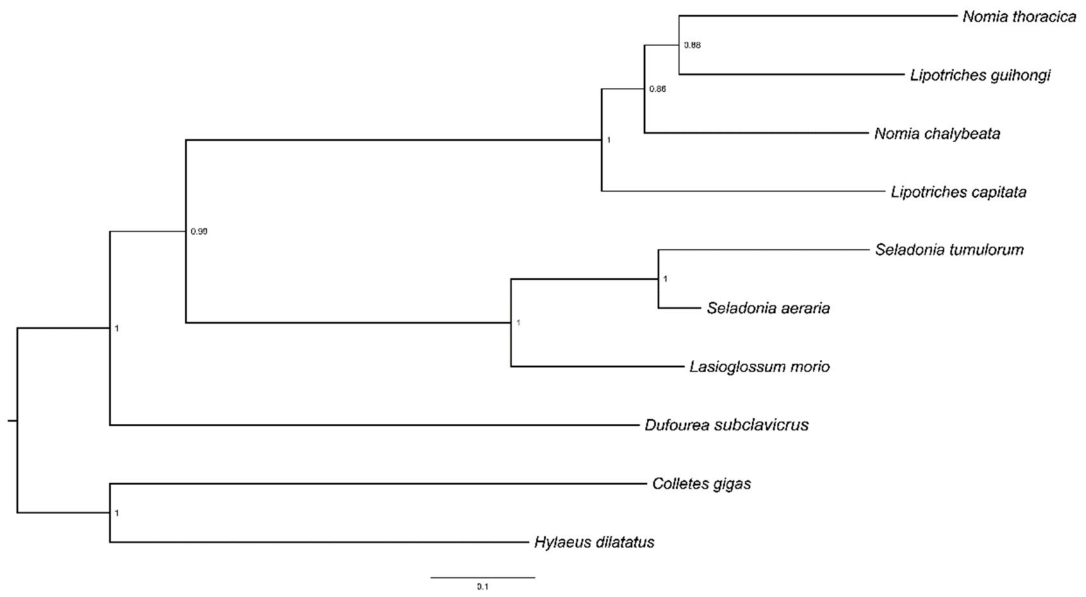

Figure S7. Bayesian inference phylogenetic tree of Halictidae based on the analysis cds_rrna with a GTR + CAT model in phylobayes. Support values on nodes indicate Bayesian posterior probabilities.

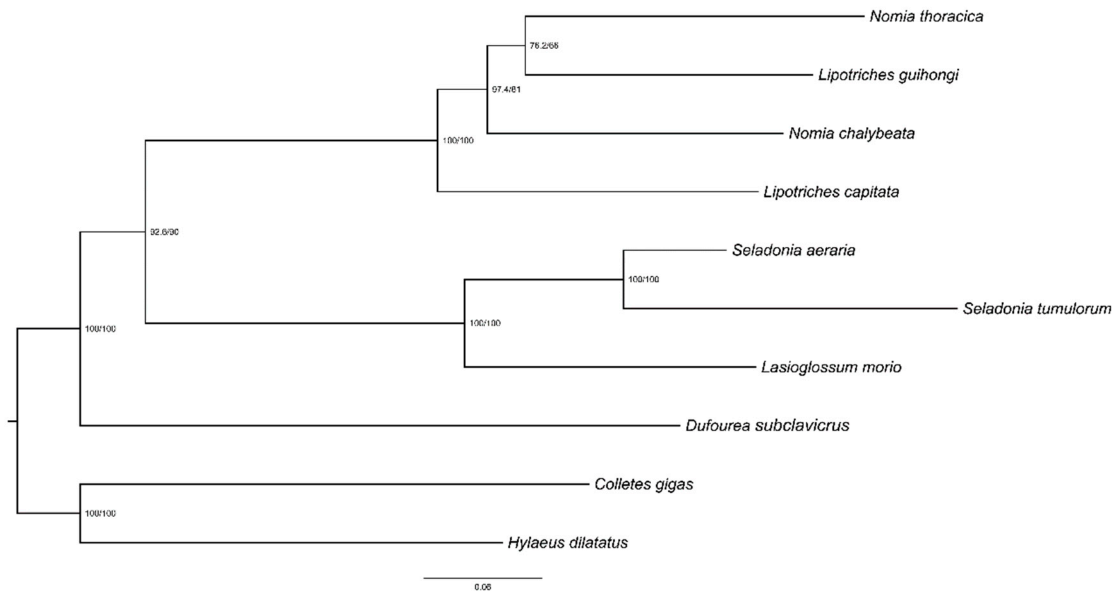

Figure S8. Maximum likelihood phylogenetic tree of Halictidae based on the analysis cds_rrna with a Partitioned model in IQTREE. Support values on nodes indicate SH-aLRT/UFBoot2, respectively.

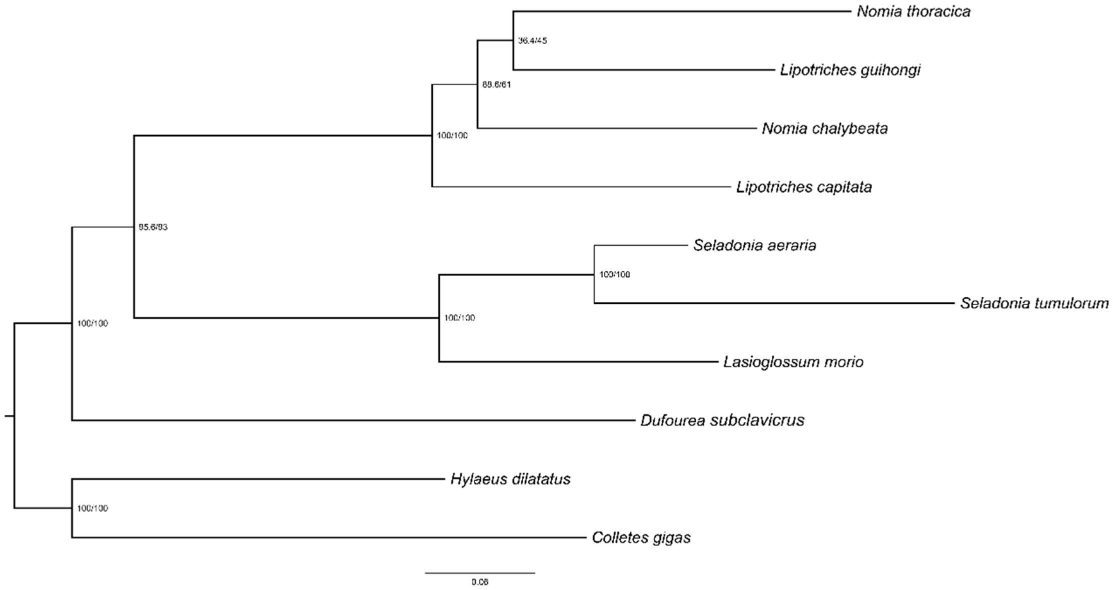

Figure S9. Maximum likelihood phylogenetic tree of Halictidae based on the analysis cds_fna with a Partitioned model in IQTREE. Support values on nodes indicate SH-aLRT/UFBoot2, respectively.
